# Stretching the boundaries of cultural policies for inclusive and sustainable urban contexts: the case of Issy-les-Moulineaux in France

**DOI:** 10.1186/s40410-022-00190-1

**Published:** 2023-01-31

**Authors:** Anna Moro, Eric Legale

**Affiliations:** 1 DAStU - Department of Architecture and Urban Studies, Politecnico di Milano, Politecnico di Milano, Milan, Italy; 2 Directeur Général Issy Media, Issy-Les-Moulineaux, France

**Keywords:** Cultural policies, Practices, Interaction, Urban regeneration, Regime-niches

## Abstract

Cultural policies can be a strategic key to shaping more inclusive and sustainable urban regeneration processes, especially exploiting the acceleration promoted through transition dynamics. The City of Issy-les-Moulineaux, for its recent and deep investment in culture, sustainability and innovation in a non-conflictual and socially homogeneous urban dense context, is a valuable demonstrator of the role and forms in which institutional cultural policies at the urban scale can produce a shift of values, acceleration or simplification of decision-making processes towards inclusive and sustainable ways of life. The presentation of examples of cultural policies and regeneration programmes aims to contribute to the identification of some innovation trajectories in this direction, looking especially at the management of cultural policies or spaces and their flexibility and openness to inhabitants. Conclusions sketch the potential of strengthening some regime-niche traits of cultural policies in Issy and creating a permanent experimental environment in the governance (and approach) to cultural policies that reshape from rigid to fluid, thus increasing the anti-fragile nature of public policies and practices.

## Introduction

### Cultural policies as urban innovators

Urban regeneration can be observed through the lens of cultural policies, considering their interrelation, which can happen at least at three levels. In the first, the relationship is due to the simple fact that cultural policies produce services and events that take place in dedicated facilities or public spaces; in the second, we can observe how cultural policies are often integrated with regeneration programs as new functions; and finally, in the third level, cultural initiatives in a broader sense can be merged and can support regeneration processes from conception to realisation and maintenance. This multiple linkage is a relevant key for understand how transition-towards-sustainability dynamics affect and link society with its settled environment (the context).

From the therethical point of view, this contribution refers to transition studies described as "[…] an emerging field of research that seeks to integrate insights from areas such as complexity science, innovation studies, sociology and environmental science to better understand large scale systemic change in societal systems and explore possibilities for influencing the speed and direction of change in these systems" (Loorbach et al. 2015, p.1), since it assumes a specific use case and verifies whether it can be described as a “regime niche”—as developed by Geels (2002) and by Grin Rotmans and Schot (2010),where paths of innovation take place. This paper refers to transition management literature as developed by the Sustainability Transitions Research Network (STRN 2019) and by Loorbach et al. (2017) and others as a framework to analyse the case and to suggest further steps not developed yet within the regime.

The chosen case deals with the articulated system and programme, rich in cultural policies and projects, of Issy-les-Moulineaux, in the metropolitan area of Paris. As shown in the dissertation, the case represents a best practice in managing cultural policies and, probably, urban policies as well. The city is in a particularly dense urban context and does not suffer muscular contractions and conflicts. On the contrary, most inhabitants are in a condition of medium/high well-being. Even though Issy policies have not been oriented according to a specific management transition model applied to the city (Frantzeskaki et al. 2018), as it has occurred in the Netherlands in the last decades (Loorbach et al. 2015), this paper aims to verify the existence of endogenous transition dynamics in the presence of quite traditional management. The hypothesis is that the given context can be a demonstrator for a potential, very effective and context-tuned approach towards transition. This belief relies on the exemplary traits of the case: the relatively homogeneous socio-cultural context, the prevailing technological and digital approach to services, and the openness and innovation promoted, as recently stressed by the pandemic. This final trait mainly concerns two aspects: one is linked to the more significant room for initiatives and authorship by the local community in general, the other is linked to a partial loosening in the ruling and governing of socio-cultural activities.

Therefore, the interest in research on the Issy-les-Moulineaux case and, in general, on the interaction between researchers and actors of urban policies, as the authors of this work are, relies on the proposition of a singular contribution on the role and forms in which cultural policies, at the urban scale, contribute to a shift of values, acceleration or simplification of decision-making processes, and promote a transition towards inclusive and sustainable ways of life and behaviours.

## Theoretical framework

To introduce our reflection, we clarify how cultural policies are a relevant access point to transition dynamics. To this aim, we point at the interpretation of two elements of the discourse: the process of transition and urban cultural policies.

*Transition* theory and its application are part of a large set of studies dealing with different disciplines as extensively examined by Loorbach (Loorbach et al. 2017, 2020) and others (Köhler et al. 2019, Schlaile and Urmetzer 2019). In this paper it is referred to generally in terms of “sustainable transition”, as used by Geels (2011) and others to stress trajectories of innovation that aim at a systemic change in the economy, society and technology, in a co-evolutive perspective, through a long-term process (Rotmans et al. 2001), instead of a gradual adjustment to reach an effective change at the level of values. Transition is crucial because it opens up possibilities when “problems appear to be very difficult to resolve […], the persistence of the problems involved may be explained by the fact that these problems are caused by processes which are firmly embedded in societal structures […] as a consequence, their resolution is bound to involve both innovative practices and structural adaptations. Such profound process of change […] we call system innovations and transitions” (Grin et al. 2010, p. 2–3). For those reasons, the fact of dealing with sustainable transition implies an “inevitable entrenchment in society and culture” (idem). This extensive idea of innovation is solidly suggested in a cultural shift of the socio-technic regimes and the socio-spatial infrastructure. Urban regeneration of a given context and the related cultural policies programme is considered one of the possible contexts of transition analysis (and application). The case uses a particular point of view. Thanks to the strong interaction with the City and functionaries in charge of many services, it is an opportunity to explore spaces and forms of innovation towards transition as they can be achieved in a traditional institutional context (in Europe, in France). The actors and the policies/practices they promoted are interpreted through a specific key that reads the mechanisms of innovation that start from circumscribed actions-arenas (*niches*) to then touch systemic conditions (*regime*) and eventually affect value (*landscape*) conditions, as developed by F.W. Geels in his work on the transition of complex societal systems and its contextualisation to urban settings with a focus on innovating practices and processes (Geels 2002; Geels and Schot 2007) as further analysed and developed with reference to urban context and design practices (Concilio Tosoni 2019).

*Cultural urban policies* are a good field of observation and experimentation on sustainable transition. This is much true, depending on the underpinned idea of culture. In this essay, we use a broad interpretation of cultural policies, both for the role we think cultural policies can have in regenerating urbanity and for the variety of elements we consider ranging from formalised policies to informal practices. The word “practices” refers to a comprehensive set of activities that can be defined as “what people do” (Crosta 2002, 2003, 2010), placing the acting subjects as protagonists. The word “culture” used to almost exclusively designate the offer of cultural practices and services in modern societies, particularly in the arts and letters, is a misnomer. This can be quite a current understanding of it in the institutional and governance domain of public administration. Culture, on the other hand, defines civilisation. It is what is common to a group of individuals, what unites them, and what is learned, transmitted, produced and invented. Thus, according to an international organisation like UNESCO: “culture can today be considered as the set of distinctive spiritual, material, intellectual and emotional traits which characterise a society or a social group. In addition to the arts, letters and sciences, it encompasses ways of life, laws, value systems, traditions and beliefs”. Culture defines the feeling of belonging to the same community. And especially at the scale of a city, cultural differences can be significant for the description and the development of a context. Considering the urban domain, we can read the manifestation of culture in a broader sense, in the materiality of the territory itself, as a deposit and signs of human action (Turri 2006). This overlapping of layers constitutes a palimpsest of multiple meanings (Corboz 1998). There are artefacts dedicated explicitly to culture: museums, spaces for art, media libraries, libraries, cinemas, etc., custodians of culture, epicentres of its diffusion, like schools for education, sports services for well-being practices and *loisir*. And then there are cultural “practices” (actions, programs, activities) and their actant and targets, protagonists of a specific cultural policy. Art, culture and education are strictly synergistic, and the practices within these domains have a strong relationship with the territories and communities that inhabit them. From a different perspective, art and culture are associated with the creative dimensions, described as the creative city (Landry 2000), the creative economy (Howkins 2013) and the creative class (Florida 2002). Landry argues that at the basis of the creative city is an idea of culture as a value, a way of life and a milieu for creativity to express. In this domain, arts and cultural activities have a different primary role since they “can foster social inclusion, provide a sense of belonging, and help promote social cohesion and reduce isolation […] participation in cultural activities instils self-confidence, pride, and personal well-being […] promote personal, community, and national identity and provide creative mechanisms for individuals to express their individuality, engage with others, and celebrate diversity” (Sdrali 2020, p.2). In this sense, culture and art can open up paths to innovation in the urban domain by tackling in-first-person engagement and action.

This paper aims to verify the existence of transitional trajectories towards innovation in the city’s regeneration field. To this aim, we analyse the case form very closely to catch the potential of emerging practices, especially by examining the organisation and the spatial dimension of cultural policies in the specific context-based situation. The qualitative/quantitative analysis framework refers to the “transition management theory” application. Transition management “is an analytical lens to assess how societal actors deal with complex societal issues at different levels, but consequently also to develop and implement strategies to influence these ‘natural’ governance processes. […] In practice, transition management comes down to a combination of developing around a common understanding of a transition challenge and a shared ambition to drive it toward sustainability.” (Loorbach et al. 2015). It is generally understood as both an analytical framework and a prescriptive policy tool (Loorbach et al. 2017; Rotmans et al. 2001). We refer to it in particular as a point of view from which we look into ongoing processes and forms of interactions associated with cultural policies which, in our understanding, are producing learning and affiliation among institutions and civic society. The analysed programme and projects (paragraph 4) are not studied in terms of their general purpose (of the urban regeneration, of the cultural facilities and policy in itself) but in terms of their experimental value, as intended in the “transition experiments” described by Loorbach et al. (2015). The experimental value, we argue here, could rely on the capability of policies to enable urban stakeholders or agencies to play a relevant role towards a sustainable transition of the urban contexts. In this way, transition trajectories are interesting since they are vehicles for inclusiveness and openness to the broader range of interests and stakeholders beyond the immediate extensor or targets. Open processes can thus stretch the opportunity to tackle many existing criticisms in urban contexts.

This specific attention to cultural programs in the urban context allows for identifying some lines of action in the envisaged perspective of systemic change. To catch the value of those programs, this paper observes city spaces and cultural policies focusing on relational dynamics, governance aspects of the process, strategies, inclusive practices and forms of uses as forms of experimentation of the transition into action.

## Methodology and interaction among different fields and languages

This paper is the outcome of dialogue between research and the applicative dimension of the city’s cultural policies, and thus reflects the origin of its two authors.^1^

The interaction between the two components took place through several formal and informal exchanges. In particular, there were five sections of discussions and shared work, distributed from July 2021 to January 2022, with the participation of the authors and other colleagues involved in the MESOC (Measuring the Social Impact of Culture-Horizon 2020) project in the first sections (one WS, in July), collaborators of the City of Issy Les Moulineaux in the core part of the work (four WS distributed in September–December), and one final specific interaction with an international expert (December).^2^

The research could benefit from the excellent knowledge and awareness of cultural policies in the urban domain of the various local stakeholders involved in preparing this work.^3^

The City of Issy provided the geographic database through the “DATAISSY.COM” portal. Additional data was obtained from institutional public databases or private ones.^4^ This part of research introduces the main features of the Issy territory, mainly investigating the inhabitants’ social profile in “Introducing the city of Issy-les-Moulineaux” Section.

The in-depth study of cultural policies related to the urban domain has been supported by rich technical documentation pertaining to policies and projects described in “Cultural policies in Issy-les-Moulineaux”and “Cultural policies and the urban domain: three different levels of observation” sections. These are public reports of the Municipality’s various initiatives, a minor part of the documentation deriving from internal studies, and still unpublished interim reports produced by the Clavim Association and made available for the occasion.

In these reports, we particularly analysed the various detailed information focusing on:

- the number and the typology of public or users of Issy’s cultural facilities, studying the targets, the type of users participating in the events, and the types of related activities, focusing on recent years, i.e., 2019 and 2020 (which show huge variations due to COVID-19 measures) and 2021 (still unpublished);
- the features of the spaces hosting the activities (internal–external/with fixed spatial structure-flexible, etc.) and the urban implications and links of the activities (their taking place in a specific area, their relation with the regeneration process, etc.);
- the aspect of communication, understanding how people are informed/involved in the cultural life of the city;
- the relationship with schools, families and neighbourhoods.

We also discuss the level of interaction of users, mainly from an empirical perspective, through interviews with the aforementioned local experts, considering the frequency of participation in cultural activities (from affiliation to participate in a specific programme up to genuine co-design of activities), how the cultural offer considers the requests and interests of its public (on-line surveys, dialogues, satisfaction questionnaires, etc.), the permeability of spaces/activities to users’ self-organisation.

We finally focussed on the quality of the offer, considering the frequency of the linkages of Issy’s cultural programme with national and international networks.

In the presentation of the case study, after a general overview of the cultural policies landscape, we present a deeper investigation of three different best practices which represent three different scales and contexts of observation of the approach to cultural policies in the urban context. The three key access deals with: the in-depth analysis of the programme, the spaces and strategies of *Le Temps des Cerises*, a best practice within the Issy panorama in “Le Temps des Cerises: a hub for learning, loisir and interaction among the community” section, a description of the significant, extensive and relevant programme and project of urban regeneration in the city in “Large urban regeneration programs and the involvement of the citizens” section, and, finally, with the various system of cultural initiatives enacted during the Pandemic by the Municipality in “System of cultural initiatives: an urgent response to the Pandemic” section. The differentiation provides an overview of tentative trends of openness and flexibility of activities or spaces that can still be further improved, showing how the special *niche* conditions of the context can offer an opportunity to put innovation to the test. The three sets of initiatives were analysed by defining the extensor, the targets, the timing and their spatial implications. Despite the differences, they shared some features: all the mentioned initiatives and projects are considered positive examples both by their promoters and by the targeted users; they are conducted in specific conditions—of open and inclusive governance, quite effective timing and an abundance of resources—that we consider to be “accelerators” of the experimental value of the initiatives. In such an environment, we argue that innovation trajectories are facilitated to emerge. The point of this paper is to define the condition of their appearance and suggest their potential intensification in order to achieve a transformation at the *regime* level, also involving the landscape sphere.

## Integrating culture, urban regeneration and technology: the case of Issy-les-Moulineaux

To understand the specificity of the case, we give a general presentation of the urban context and of the way it is inhabited in “Introducing the city of Issy-les-Moulineaux” section, describing the overall cultural approach of the City in “Cultural policies in Issy-les-Moulineaux” section.

### Introducing the city of Issy-les-Moulineaux

**Fig. 1:**
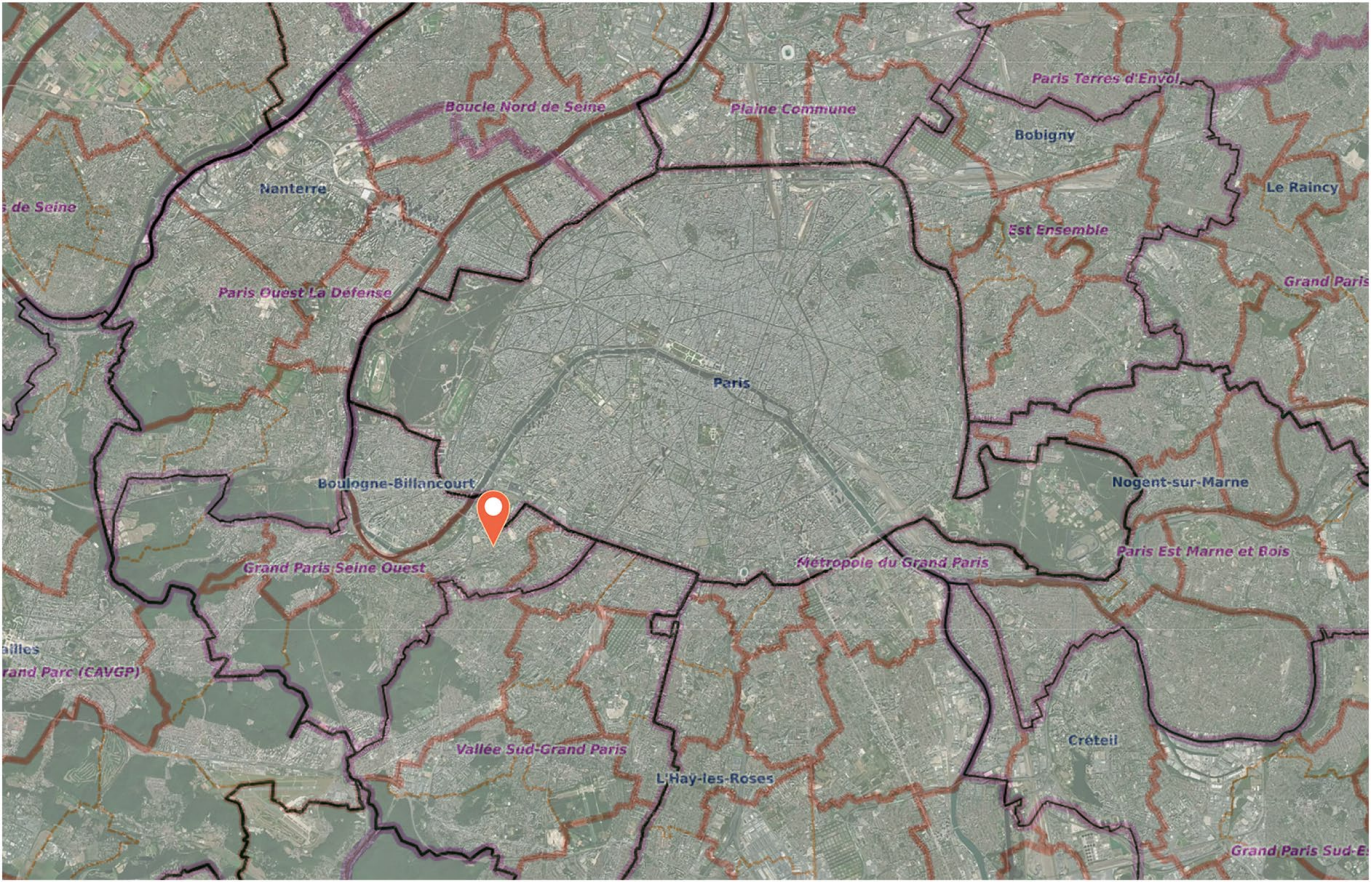
Localisation of the municipality of Issy-les-Moulineaux. Source: Authors elaboration from DATA ISSY.COM—Municipality of Issy-les-Moulineaux

The City of Issy-les-Moulineaux is part of the Metropolis of Greater Paris (Fig. 1). As a former productive site, Issy experienced very different seasons as the economic and labour sectors transformed. After the dismantling of industries and productive platforms started in the 70/80s, an extensive effort was carried out to change the production and go towards technology and innovation.

The City has now become one of the most economically and demographically dynamic cities in the Hauts-de-Seine. After dropping to about 47,000 inhabitants in 1975, the city today has 70,000 inhabitants and 72,000 jobs and stands out for its socio-economic dynamism.

International companies have replaced the former productive sites.

“Coca-Cola, Microsoft, Accor Hotels, Sodexo, La Poste, Icade, Orange. At the southwest gates of the capital, Issy-les-Moulineaux is always attractive for the headquarters of large international companies.” Le Parisien, 6 June 2016 (*translation of the authors*)

With over 720 businesses/artisans and 800 active businesses, more than 50% of which are in the communication and information technology sectors, the City has created an efficient economic framework. Since the mid-90s, Issy has embarked on information and communication technologies and has become a recognised reference in this field both in France and abroad.

“The Ile-de-France Region has launched the platform “Ile de France Smart Services”. Issy-les-Moulineaux has been one of the first Cities to have signed this agreement. The implementation of these services is the result of an unprecedented partnership approach around data between public and private actors, as it also represents a common data portal including datasets and data visualisation tools” (Acar et al. 2020).

In this context, new jobs are created, new families are attracted and the current profile of Issy’s population is quite impressive.

Nearly 65% of the population is under 45 years old, and over 39.1% of them are under 30. Around 12.2% of the population is over 65. Families represent 55.7% of the total number of households, and middle-ranking and senior executives represent 51.4% of the working population aged 15 or over.

**Fig. 2:**
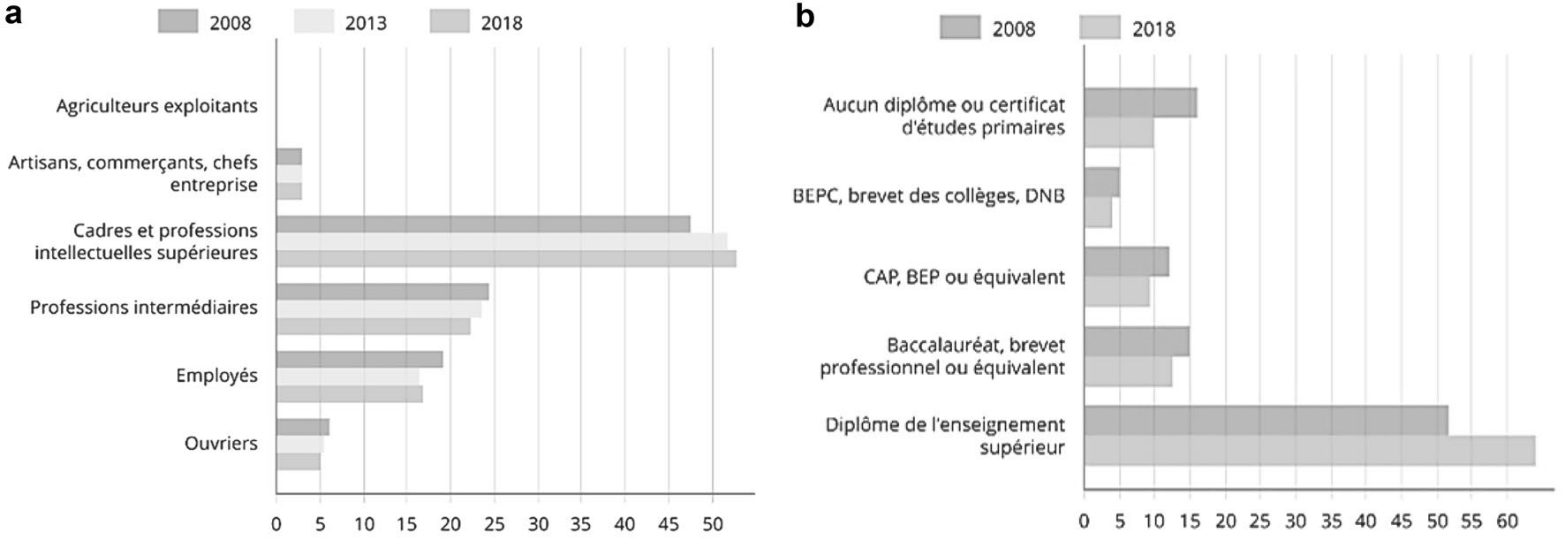
**a** Variation of Jobs by socio-professional category (2008–2018). **b** Highest diploma of the out-of-school population aged 15 or over (%). Source: Insee, “RP2018 exploitation complémentaire lieu de travail”, date 01/01/2021

Regarding employment, we can see that 52% of the population is an executive or a higher intellectual profession, while France’s average is 9% (Table 1). This trend is positive from 2008 to 2018 (Fig. 2), and 60% have a higher education qualification, while France’s average is 29% (Table 2).

**Table 1:** Jobs by socio-professional category in 2018. Source: Insee, “RP2018 Exploitation complémentaire lieu de travail” date 01/01/2021

	Number	%
Together	52,876	100.0
Farmer operators	0	0.0
Artisans, traders, business leaders	1603	3.0
Managers and higher intellectual professions	27,903	52.8
Intermediate professions	11,771	22.3
Employees	8926	16.9
Workers	2673	5.1

Housing standards are relatively high standards; important investments have recently been made in sustainable approaches and qualitative solutions. The price per sqm^5^ is relatively high and comparable to Boulogne-Billancourt and almost Paris, and higher to the area’s average price. At the same time, there is a fixed percentage of social housing at about 20–25% in recently developed sites. Issy-les-Moulineaux is also a pretty safe place, where the crime rate is minimal compared to the metropolitan area and the average of France.^6^

This picture confirms the specificity of the southwest metropolitan area around Paris, known as the “Parisian great west” (*Grande Couronne and adjoining administrative departments*) characterised by middle-class households. If a distinction is to be made, it is between the higher and lower middle classes.

“According to the social milieu and the residential proximities but also the residential and daily mobility strategies, a fracture takes shape between a fraction of the middle class assimilated to executives belonging to a “high middle class” and the “low, middle class”. The latter being composed of forepersons and supervisors, often assimilated to white-collars and labourers, often constrained to become owners further away from town. Statistical analysis [...] highlight the integration of the middle class in the peri-urban space, making it a space of resources, family proximities, social ascension and security through homeownership. Yet, these analyses also highlight the weakening of a fraction of the middle class caused both by the rapid increase of property value in the closest suburbs and the increasing cost of mobility. While household adaptation strategies often favour proximities, local governments are seeking new forms of peri-urban development” (Aragau, Berger, Rougé, 2019).

In such a context, where Issy-les-Moulineaux is part of this “space of resources, family proximities and social ascension”, we are interested in understanding which features and roles cultural policies play.

### Cultural policies in Issy-les-Moulineaux

Three ingredients describe the efficiency of the city’s cultural infrastructure. First, the material facilities (buildings and spaces) often function as hubs of interaction among people thanks to their governance and organisation; second, an efficient network of interlaced services that is constantly creating and reinforcing a solid community over time; and third, the various offer of cultural experiences, where technology and digital aspects play a relevant and recursive role, is managed as a sort of city label.

“Since 1995, the city of Issy-les-Moulineaux has pursued a proactive policy to support the development of information and communication technologies, adapting services support the development of information and communication technologies, adapting the services offered to residents or increasing the number of innovative services such as payment for parking by mobile phone or the Daily Life Card. […] Issy is distinguished by a particularly dynamic economic fabric. 57% of its companies are in the technology sector.” (André Santini 2004)

**Fig. 3:**
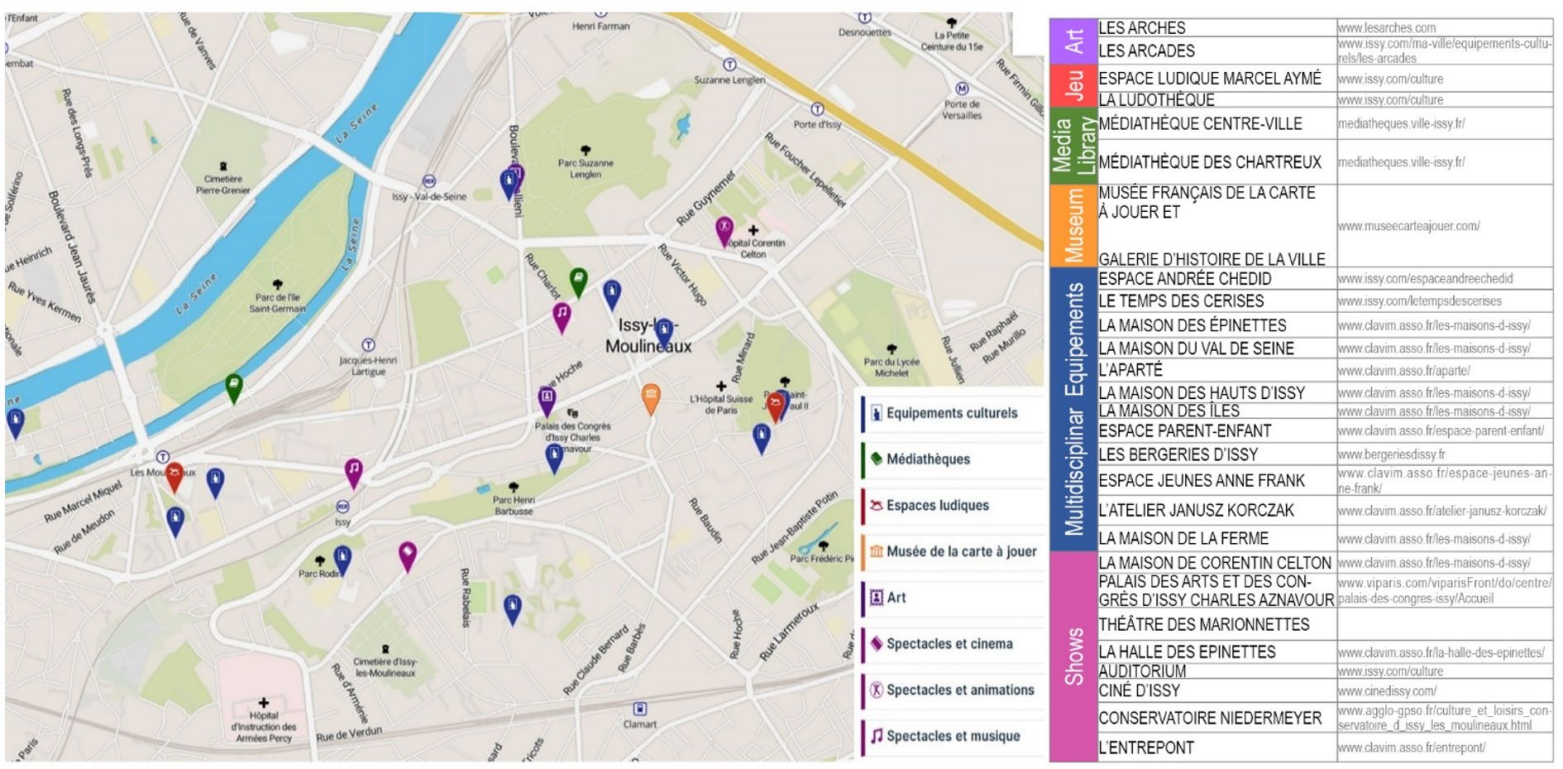
Localisation of the cultural facilities by typologies. Source: DATA ISSY.COM—Municipality of Issy-les-Moulineaux

Issy-les-Moulineaux has one convention centre, three media libraries, one museum, one art school, one music academy and a nursery of amateur musicians, workshops of artists from all over the world, two playgrounds and one digital creation centre. In town there are about 27 cultural organisations offering a vibrant programme to the people of Issy all year round. The City’s strategy is to provide all residents with access to a diverse and high-quality cultural life. The organisation and management of cultural equipment mainly rely on one public very performative association, CLAVIM (*Cultures, Loisirs, Animations de la Ville d'Issy-les-Moulineaux*), a fundamental “institution” for the inhabitants. Among the most relevant cultural facilities in the city, we can quote the mediateque “Le Temps des Cerises”, the space for music and concert “Réacteur”, the “Espace Andrée Chedid” for families devoted to philosophy and poetry, the “Halle des épinettes”, a movie club that is an educational pole dedicated to images, and finally the Atelier Janusz Korczak which deals with theatre and performances, and many others (Fig. 3).

Many of these offer a service for young people. There are no specific formal written agreements for relationships with local schools. The link between the national education system and the activities provided by the City and by Clavim is strong. It is formalised directly with the Ministry of Education through a yearly procedure. At the beginning of the school year, Clavim submits the “academic inspector”, a ministerial supervisor for the whole scholastic primary cycle, proposals for activities such as guided tours, expositions, laboratories, etc.^7^ This mainly occurred in the pre-pandemic phase, while in 2020–21, activities were primarily organised within the schools. Clavim also manages the extracurricular activity of the “Wednesday afternoon” (when French schools are usually closed), and holidays, when children are addressed to recreation centres or leisure centres placed inside the various schools in Issy-les-Moulineaux. The association is engaged in interacting with secondary education as well. The four Colleges and one High School are involved through FabLab laboratory (3D printing, coding, robotics) and the digital museum (this activity became more difficult during the Pandemic). In the end, facilities and the various programmes offered by Clavim welcome many schoolchildren throughout the year, constituting precious support in parenting, opening up to new themes, and experimenting with culture and technology.

The investment to reach such a standard is high and is gained partially through specific political choices.

“We are also one of the four or five big cities that have neither municipal police nor surveillance cameras. If we had a municipal police force in the city, a police force of eighty people, with the equipment that goes with it, it would cost almost 10 million euros. Today, the subsidy paid to the leisure and entertainment centre of the City of Issy-les-Moulineaux (Clavim) by the City is 5 million euros, and Clavim has 2 million additional revenue; its overall budget is therefore 7 million euros. As a result, 6500 young people and adults attend the Clavim for one activity or another, about 15% of the Issy population, with about fifteen facilities, twenty-four leisure centres, Entrepont, la Halle des Épinettes, a youth space, a one-stop-shop for young people… it’s considerable. Political choices are budgetary choices” (Santini 2004).^8^

The idea of the Municipality is to go even more in this direction and equip every sub-area of the city with one facility dedicated to culture,^9^ following the well-known “15 min city” policy advocated by the academic Carlos Moreno (Moreno et al. 2021).

## Cultural policies and the urban domain: three different levels of observation

The cultural environment of Issy-les-Moulineaux offers an opportunity to look into the three dimensions of the relation between cultural policies and urban space, as mentioned in the introduction: the physical dimension, i.e., equipment, the functional one, i.e., regeneration programmes, and the interactive dimension, displayed at various levels.

### “Le Temps des Cerises”: a hub for learning, loisir and interaction among the community

**Fig. 4:**
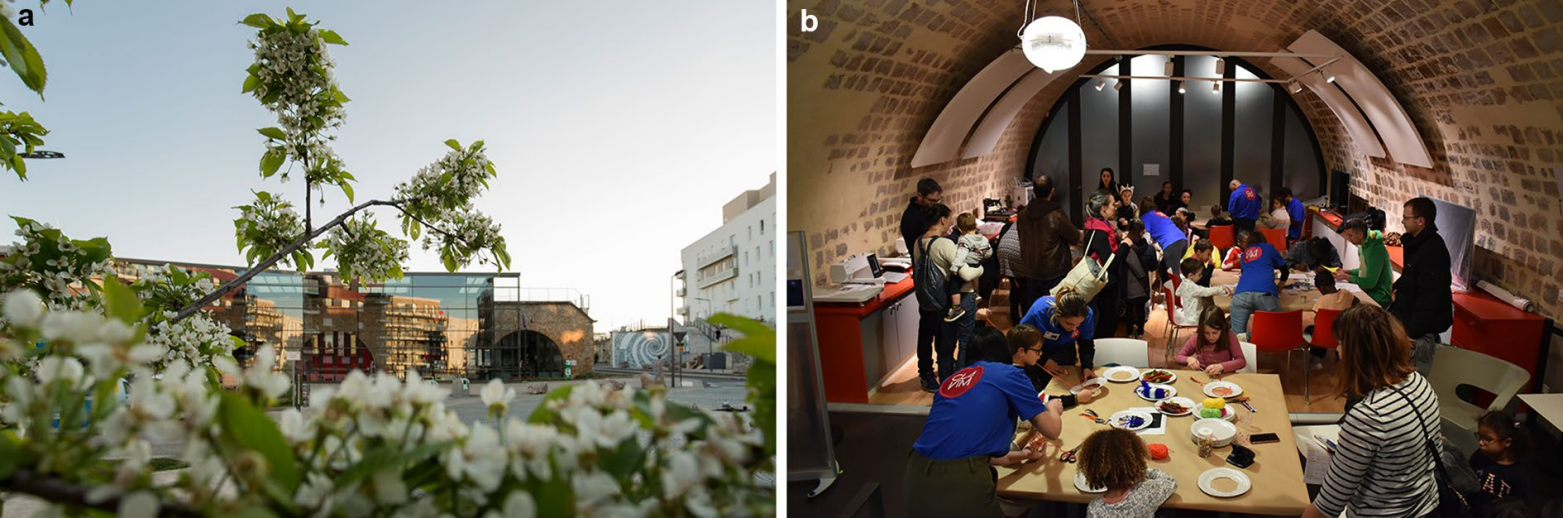
**a**, **b** The exterior of “Le Temps des Cerises”, on the Promenade du Verger, and interior space for activities. Source: CLAVIM association—Ph: DavidPhotographe

In the first case, a very successful example is provided by the cultural centre “Le Temps des Cerises”, named after the famous popular song by Jean-Baptiste Clément, emblematic of Paris and the region. The building, located in the eco-district of Fort d'Issy, very close to the site of the City of Issy-les-Moulineaux, was rehabilitated in 2014 with an attention to maintain the historical feature of the Fort, combining the contemporary architectonical language and the memory of the pre-existing site (Figs. 3, 4a, b).

**Fig. 5:**
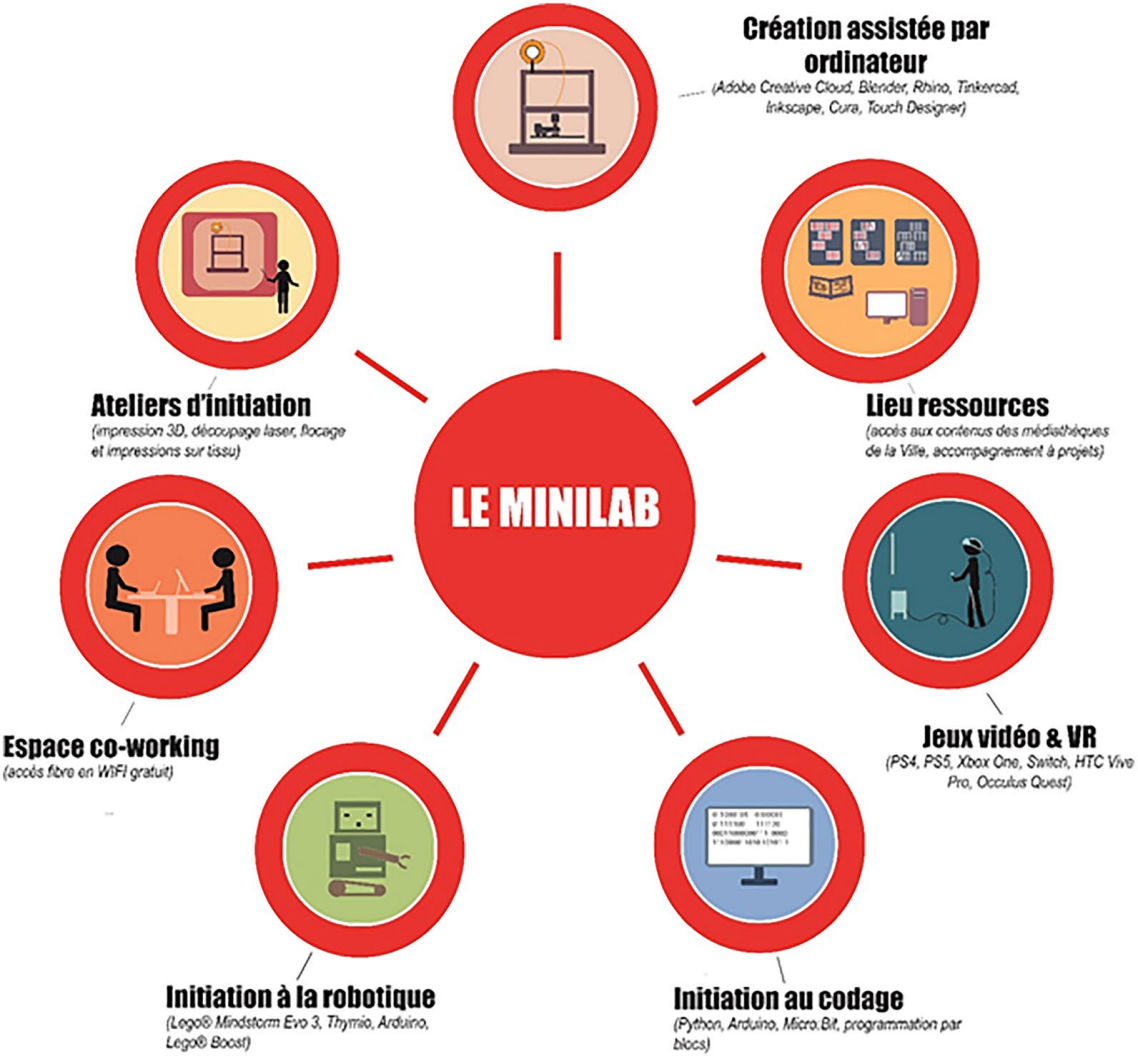
Example of different activities provided by the Minilab inside “Le temps des Cerises”

The Clavim association, which manages it, describes *Temps des Cerises* as “an equipment of multidisciplinary activities, cultural discoveries, playful, digital, and scientific experiments, meetings, and exchanges for all audiences”. *Temps des Cerises*, with its virtual installation of art masterpieces, is part of the *Micro-Folies* network. It has been the first in the Hauts-de-Seine area to join the national network and host a cultural device that promotes access to art through digital tools and innovative learning approaches. The place is part of networks of institutions and valuable cultural spaces and programmes, as a resource for local schools and families. Diverse activities are integrated within the same building, promoting a rich programme of initiatives devoted to multiple public targets (Table 2). The Minilab, for instance, is a flexible space capable of integrating culture with educational training, experimentality and entrepreneurship (Fig. 5).

**Table 2:** Spaces and activities of “Le Temps des Cerises”. Source: CLAVIM aasociation

1. THE MEDIA LIBRARY (MEDIATHÈQUE)	Le Temps des Cerises has a 120 sqm media library intended for all audiences, connected to the network of the two other media libraries in the city. This space is a place of service (borrowing documents) and acceptance (an area for reading and studying). Several digital services are available to you (machines, online catalogues, return terminals, etc.)
2. THE MANGATHEQUE	More than 200 collections are available on-site or by loan. A partnership has also been established with the Maison de la Culture du Japan in Paris to discover multiple facets of Japanese culture. This room is also the subject of a collaborative operation project
3. THE MEMORIAL WALL	To enhance this high place of Issy's past, a memory wall, designed by the French Museum of the Playing Card and Issy Media, tells in interactive form the history of Fort d'Issy and the Paris Commune, in particular the events of the “Terrible Year” (1870–71)
4. THE PAUL RICOEUR ROOM	This room is intended primarily for readers and students. It can also be used as a conference room, workshop, or show. Since September 2016, it has bore the name of Paul Ricoeur in homage to the philosopher. A specific fund allows audiences to approach its philosophy through reading, workshops, screenings, or conferences
5. THE JACQUES HINTZY ROOM—MINILAB	This space is devoted to fun and digital activities with the possibility of playing on-site (consoles, virtual reality) or participating in workshops or events throughout the year (3D printing, coding, etc.). It is located in the Jacques Hintzy room, inaugurated in 2014 on the 25th anniversary of the International Convention on the Child’s Rights, in tribute to the former President of UNICEF France from 1999 to 2012
6. THE AUDITORIUM—DIGITAL MUSEUM	This space can accommodate up to 50 people for live shows, puppets and object theatres, concerts, film screenings, conferences… It also hosts the Digital Museum as part of the Micro-Folie project
7. COLLABORATIVE WORKSPACE	This new shared space was completely redesigned in 2021 to allow students and residents to work in a friendly environment and take full advantage of all the activities deployed during the year at Le Temps des Cerises
8. THE VERRIÈRE AND THE CONVIVIALITY CENTER	This modular space can accommodate different exhibitions and events depending on the schedule. It also serves as a reading and discussion area and has a press corner, an information point, and a book trunk

Considering its significant role in the perspective of culture as a driver of societal transition, *Le Temps des Cerises* is an example of learning and inclusivity. The association is expanding its role, opening up spaces for independent activities organised by the inhabitants and inventing innovative programmes that use digital technologies to attract and involve youth (but without forgetting adults and the elderly). In this way, *Le Temps des Cerises*, with this extensive range of opportunities, can be perceived as a space to shorten the distance between learning/innovation/culture and everyday life. Thanks to technology, cultural is easily integrated into ordinary life, and the inclusiveness and sense of participation and belonging are strengthened. The cultural initiatives are treated and proposed by learning and enabling awareness about the history, sustainability, arts, etc. The role of technology is to help share art and knowledge more efficiently.

### Large urban regeneration programs and the involvement of the citizens

Considering the relation between cultural initiatives and the transformation of the city, we can observe the effort of the Municipality to communicate its ongoing regeneration programmes, especially through digital devices,^10^ such as digital models of urban development projects, to better inform citizens about the future projects, is one of the main features of the interactive policy implemented in this context. Integrating citizen activism and ideas is part of this strategy and approach to the governance of the City. Participation has a relevant space in the city’s discourse through different devices.

**Fig. 6:**
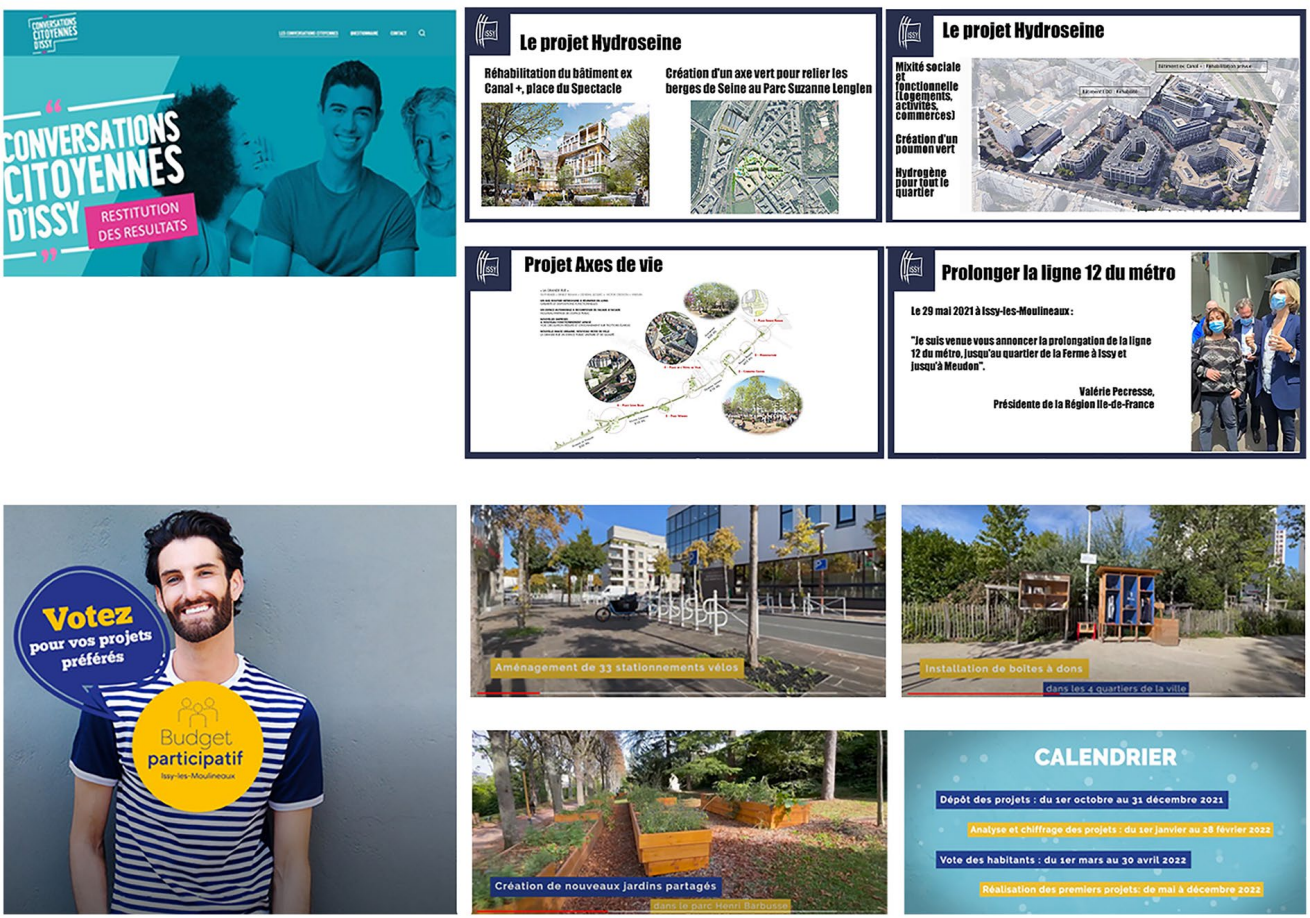
*Conversations citoyennes d'Issy-les-Moulineaux* and some ideas that have been proposed (up). *Budget participatif d’Issy-les-Moulineaux* and some of the results in terms of urban fornitures and regeneration of the public space (down). Source: authors elaboration from Issy-les-Moulineaux web site public documents

The Municipality has recently launched a new tool. *Conversations citoyennes d'Issy-les-Moulineaux* is an open online forum where citizens pose questions. The survey monitors the satisfaction of living/working in Issy-les-Moulineaux, the priority measures to be developed by the city, solutions for climate change, etc. The consultation started in November 2020. The response of the Issy population was relatively high^11^ in terms of the public. The survey investigates the current conditions (90% of respondents are satisfied with living or working in Issy-les-Moulineaux) and inhabitants’ ideas for the city’s future development. Quoting some outcomes,^12^ people underline the importance of working on public green space issues, the improvement of public transport, the interest in a cycle plan, the improvement of retail and proximity services and commerce and the implementation of the physical surveillance of the city (Fig. 6-up).

Since 2002, Issy has developed a participatory budget (*Budget participatif d’Issy-les-Moulineaux*) after returning from a visit to Brasil, where the Municipality studied the strong local interactive culture and its various initiatives. The City uses the participatory budget to fund local industries and increase their visibility. The amount of funding is moderate; however, it often allows the realisation of independent initiatives for public space regeneration and social programmes to help and support fragile categories. The attention to public space and civic engagement is also emerging through the attention to urban initiatives such as gardening,^13^ pedestrianisation, and mobility policies^14^ (Fig. 6-down).

The regeneration of the urban environment with an attention to the quality of life, implementation of technology and innovation of solutions in a sustainable future perspective (Veltz et al. 2018) is prioritised and balanced with other budgetary objectives also in significant primary interventions in the city.

Issy-les-Moulineaux has changed its face in a few years thanks to a proactive and balanced urban policy that has made it possible to build housing and reclaim public spaces. Between 1995 and 1999, the surface area of green spaces increased from 42 hectares to 52.5 today (or 13% of the municipality’s territory against 9% on average for the cities of Ile-de-France).

Many former productive sites were transformed in the last 20 years with considerable effort and investment. The city of Issy-les-Moulineaux has undertaken the realisation of large-scale architectural projects to connect the city to the river Seine and its banks. The main urban regeneration has created new neighbourhoods and districts such as Quartier des Portes de Seine (1994–2011), Usine d’incinération «Isséane» (2003–2007), Bords de Seine part 1 (1999–2007), Aménagement de la ZAC des Chartreux (2006–2011), Bords de Seine part 2, called Ecoquartier des Bords de Seine (2008–2015), Vallée Rive Gauche: regenerated by the Conseil Départemental (2011–2017), Tri Postal (2012–2015), Port d’Issy (2012–2017), Pont d’Issy (since 2014).

One regeneration project that recently acted in this direction is the *Eco quartiers Bord de Seine* (2015), in substitution of an ancient plant (the TIRU, *Traitement Industriel des Résidus Urbains*) stopped in 2007 to be replaced elsewhere.^15^ The area is one of the last large productive sites left. The specificity of the realisation is the environmental performance, where buildings and homes have low energy consumption, thanks to bioclimatic design, reinforced insulation, high-efficiency equipment and renewable energies (solar thermal, heating network, etc.). The urban transformation produces a mixed-function neighbourhood that hosts company headquarters and offices for firms mainly devoted to digital development and communication (Microsoft, Cisco, Bull, Marie-Claire, etc.).The regeneration has also provided housing and ensured the quality of the public spaces by re-joining crucial green space in the surroundings (the parc of Ile Saint-Germain). We can read on the dedicated web page of the programme that “[…] the district today offers a preserved living environment, in which functions are mixed and inhabitants, whatever their standard of living and their age, live side by side in harmony […]”,^16^ and in a video interview, the responsible of the project (Philippe Barrau)^17^ underlines the high-quality standards in terms of public spaces and cycle lane where on the 3.5 hectares, half of the public spaces have been dedicated to pedestrians and cyclists.

**Fig. 7:**
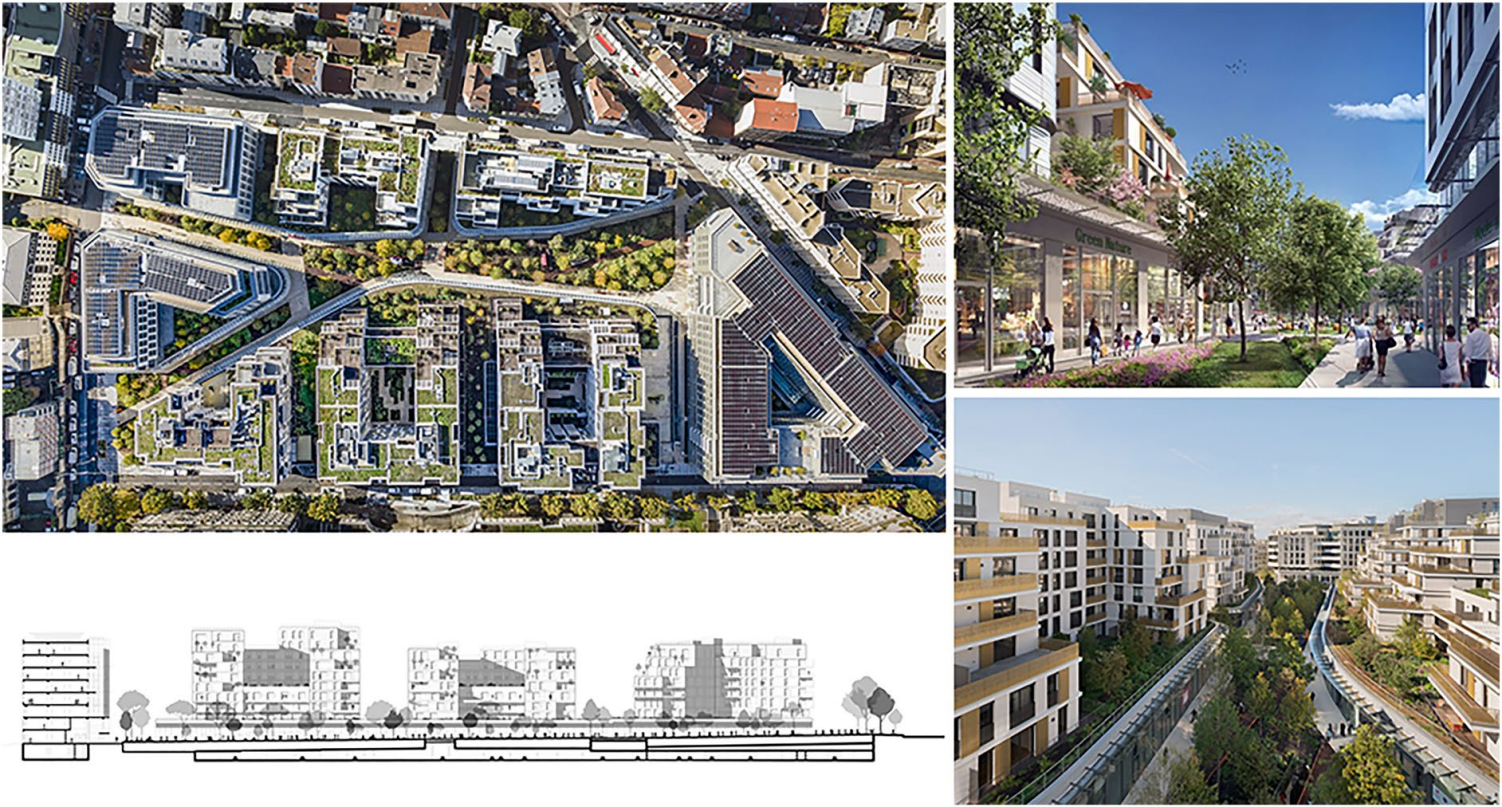
"Issy Cœur de Ville" urban regeneration sites in Issy-les-Moulineaux. Source: Authors elaboration from https://www.archdaily.com and https://www.issy.com/coeur-de-ville

Another example is the future neighbourhood of *Issy Cœur de Ville* (Fig. 7) shares the same direction, with a new masterplan and planned operations that will have a strong impact on innovation and digitalisation of services as well as on ecology-sustainability-quality of life within the urban regeneration programmes. The objective here is both to accelerate the digital dynamism of the city, also linked to other large contemporary programmes,^18^ and complete the local offer in terms of cultural facilities with structures for early childhood, schools for ten classes, shops and boutiques, a UGC cinema and restaurants. The expectation is to create attraction at the metropolitan level across Greater Paris with 300,000 expected annual visitors, benefiting from the digital-art exhibition spaces and co-working spaces. As declared by the City:

“a 1500 sqm creative workshop will be set up in the heart of this eco-district with the ambition of being an emblem for the city of Issy-les-Moulineaux. This unique place, crossing point and meeting place aim to offer a new experience adapted to the expectations of all users, residents, businesses, schools and associations. The result of the hybridisation of the best in real estate, the future space will respond to new societal and technological challenges” (7 September 2021, “Projet Issy Cœur de Ville: connecté par nature”, www.issy.com/coeur-de-ville).

The regeneration will focus on creating a veritable green connection to the other green areas of the site. At the same time, the neighbourhood will be served by a smart grid through the “IssyGrid” project. Issy Grid is an operation that intends to reduce the carbon footprint of cities, integrating new technology for the energy production and consumption of the area.

“The city’s future project is “Issy Cœur de Ville”, and its ambition is to create a smart district. It will be a natural, pedestrianised space, surrounded by a 13,000 sqm urban park. The district is looking to pursue the BiodiverCity label, rewarding efforts in terms of biodiversity, as well as the Well for Community certification, recognising work to improve health and wellbeing” (https://www.intelligentcitieschallenge.eu/cities/issy-les-moulineaux).

### System of cultural initiatives: an urgent response to the pandemic

And finally, we describe the third group of initiatives which depict an extraordinary effort to link culture in the city with its inhabitants. The Issy community has partially lived the adaptation to the pandemic as a way to innovate the cultural offer and approach. Like almost every city, the open space was an incredible resource to reframe the cultural offer.

Indeed the pandemic has brought cultural life to a halt. But it is interesting to see the imaginative work of the different structures of Issy-les-Moulineaux and the initiatives taken to maintain a quality cultural offer.

Suppose the pandemic was an opportunity to test and deploy a “click-and-go” service in most French media libraries and businesses. In that case, the Issy-les-Moulineaux media libraries have been offering this service since 2015. In 2020 the number of reservation remained the same as in 2019, and it has risen since the start of this year. The “click & collect” service has been available for toy libraries since May 2020.

A wide variety of remote meetings and online content has been devised: live concerts as part of the “Window on the World” cycle and “Sundays in melodies”; retransmission of conferences; shows for young audiences, uploading of old audio versions; meetings in English on Sunday morning for “Books and Breakfast” and on cultural news on Sunday afternoon during “Discovery rooms” or among teenage readers in the very famous Club de Mme Pince. A playlist on “Issy.TV” is dedicated to media libraries to access numerous content. The “mediatheques.ville-issy.fr” site is enriched daily with reading tips and musical playlists and allows you to find all this programming.

The school exception prevailed with the organisation directly in the classes of workshops initially planned in media libraries: initiation to lyrical music, world music and instruments.

The digital alternative was implemented at the Espace Andrée Chedid from March 2020. It mainly resulted in posting cultural content online, video-conference events and production podcasts. As of 20 March 2020, video poems were published on the space’s Facebook page. Over 2000 people have viewed the videos. Since then, the team has made films to break through the confinement walls and provide audiences with rich programming; to support as much possible a well-established cultural sector. A real challenge, the haiku fair was maintained wholly digital at the beginning of December. In total, more than 21,000 views or participation in events were recorded.

“The Wonders of the Museum” is a new cycle of videos to present their permanent journey through a selection of their masterpieces. Revolutionary period tarot or heraldic card game from the seventeenth century, these videos demonstrate the playing card's evolution in connection with France’s history or its devious uses such as the typographic office. This cycle is also open to the Museum’s sculptures, such as Rodin’s.

Podcasts have been accompanying the Museum’s exhibitions since 2019 with “Architectural Landscapes” for Raymond Depardon’s photographs of emblematic buildings in the City. The “Cartomancy” exhibition was deepened by the eyes of specialists who, during five episodes, explored it through the magic of cards, rituals in hospitals or the fascinating stories of the great fortune-teller of All-Paris in the 19th Century. It was also possible to browse the exhibition through its podcast tour when it was only physically visible for 2 months.

Since October, the Arcades have been remotely offering their “curiosity from elsewhere” cycle lectures. “The sacred geography of Nazca”, led by Aicha Bachir Bacha, teacher-researcher at EHESS, has recorded more than 700 views. Sophie de Saint-Phalle gave a presentation on the presence and visibility of Latin American women in the world of contemporary art, a video viewed nearly 600 times. In 2020, confinement and distance education had not prevented all the students in the preparatory class for the Arcades from integrating the prestigious French or European art schools (ENSBA, ENSAD, HEAR, The Hague, etc.). This year, as part of the maintenance of teaching in schools, the courses of the preparatory class of the Arcades have been maintained since the start of the 2020 school year and those of the Conservatory for minors. In compliance with health constraints, students follow classes and carry out workshops and workshops. Since January, courses for adolescents and children have been able to resume. The workshops for the general public are organised remotely.

Since the first confinement, the Reactor with CLAVIM has mobilised alongside the city of Issy-les-Moulineaux to allow the continuity of its artistic and educational actions.

## Culture for the city of Issy-les-Moulineaux: how is it tackling transition in the urban domain?

The scrolling through cultural politics and projects promoted by Issy-les-Moulineaux has provided an extensive overview of situations and issues connected to cultural politics and the regeneration of the city. A first general take-off can be done, and then two specific dimensions where transition trajectories emerge will be pointed at.

In general, we have seen that many of the initiatives promote highly innovative content. We have also observed that cultural programs—as well as the forms of management and activation of the dedicated spaces—are highly formalised within specific protocols and procedures. Protocols are often not necessarily local, sometimes referring to a higher rank (ministerial), as typical for France. We have shown Issy displays a cultural offer that is attentive to different targets. Even the approach to technology and digital is skilfully declined to be accessible. The panel of activities is so rich that nothing seems uncovered. The local public actor shows a remarkable ability to network and is present at the national and international levels. And its perspective is undoubtedly well represented in the article.

Then, two significant aspects of transitional dynamics are clearly expressed in the recent policy approach of Issy, related to trust and affiliation on the one hand and loosening and flexibility on the other.

### Trust and affiliation

To introduce how the role of trust is emerging, we can consider the aspect developed in Issy’s policies of including intangible elements in the definition of the management of cultural services. This is easily recognisable in leisure and sports typically offered to young people where relational attitudes like trust and loyalty are at the core of the offer of Issy, high in performance but also vehiculated values through services. Specific additional investments in the relationship between institutions and people are reflected in the interest in hiring new local expert trainers already integrated (or integrable) within the local services. This guarantees an increase in trust in institutions, promotes social cohesion, and can ultimately positively affect the proposed educational, cultural, sports and leisure programs. In addition, such a fiduciary approach is intended in a cumulative and constructive form over time. To quote the mayor of Issy-les-Moulineaux: “thus, each time, we prove that working in partnership and network is a good idea. We combine synergies and then it happens spontaneously” (Santini 2010—translation by the authors). In this way, we have seen, through interviews with local authorities and from user’s questionnaire/report (by the Clavim association), that cultural facilities are often perceived as meeting points for children and families, and that recursive events promote loyalty and a sense of belonging to the places and even to cultural practices, which became routines.

### Loosening and flexibility

The high functionality of the Issy-cultural system shows a sort of counterbalanced in a partial rigidity of the procedures and the interaction with the users. The hypothesis is that the city’s general organisation and management of cultural policies could benefit from undetermined or semi-structured spaces of action. In this direction, the management and performativity of the system have recently received some stresses and inputs. It is partially due to a reinforced solicitation for a break in current paradigms pushing towards transitions of current socio-economic systems, originated by the criticalities of the pandemic. Among others, one theme in Issy’s cultural policies stands firmly: it is the interest in interaction with inhabitants and continuous research in letting local values arise alongside technological dynamism. From the words of the various administrators met, the city, indeed in some controlled space dimensions, seems interested to be contaminated by a more open attitude and stronger experimentation. For instance, talking about *Le Temps des Cerises*, the facility is oriented to attract the public both from the central equipped eco-district (with a relatively high socio-cultural level although with around 25% of social housing) and the close, socially-mixed neighbourhood *Quartier Épinette*. *Le Temps des Cerises* pushes its cultural role, helping shorten distances between different social environments. And the intention is to open up more in this direction. In general, the CLAVIM association is interested in conceiving cultural spaces as places for versatility and openness in multiple orders, as the “*tiers-lieu culturel*” model. They would like to support more and more people in being actors in their projects. *Les* *Temps des Cerises* is already hosting, every year, a number (about 5 or 6) of local associations devoted to embroidery, music, environment, a “participatory coffee”, etc. Clavim and the NGO Department of the City, the subject that manages the associations in town, have signed a general agreement to facilitate and organise this cooperation. And in this way of managing the relationship, they are satisfied. The attempt to engage people more directly by opening up the space (the laboratories) is in place, but some adjustments are on the run. The argument is the level of freedom/the need for rules that have to be previously defined to clarify the parties' responsibilities. The more recent Coworking experience, where *Les* *Temps des Cerise*s gives space for people to work and provides specific equipment, is consolidated and can grow. As stated by the director of Clavim, this is “an approach that needs to be amplified, in the spirit of a cultural third-place” (from an interview conducted by the authors, November 2021).

Those two aspects open up a possible working key to opening up cultural policy processes: finding direct linkages to everyday life. Cultural, artistic and educational experiences can be observed from the point of view of the proposing institution but also of the individual participant, more or less actor and agent of cultural *practice*. The presence, the awareness of being agents within the exercise of a practice, strengthens the cultural role of the practice itself. And this makes sense if we agree that the meaning of culture is the all-encompassing one set out in the introduction of the contribution. On the one hand, this presence and awareness refer to the so-called sense of “human agency” (Bandura 1986) of the people in the actions they perform. The concept of human agency, the cornerstone of social cognitive theory, can be defined as the ability to act actively and with a transformative attitude in the context in which one is inserted. This human function, which concerns both individuals and groups, operationally translates into the possibility to generate actions aimed at specific purposes. Participatory artistic practices, co-design, and first-person action experience in the city are closely linked to this concept. Art, on this front, is understood as a form of qualification of subjects to public life, for the well-being of the individual, with an increase in the quality of life linked to the significance of one's actions within a collective interest or community. This produces a sense of immediate fulfilment of one's activity that integrates people through relationships (and trust) by rooting them in places they will want to take care of. In this perspective, the current themes of implementing a systemic change toward an inclusive and sustainable society are declining concerning the lives of citizens. It is very well known, for example, that at the European level, the issue is crucial in the discussion of the New European Bauhaus, linked to the "European Green Deal", which identifies the field of interaction between citizens, experts, institutions and businesses, as well as the protagonism of individuals as a priority to stimulate innovation (Bason et al. 2021).

In the end, we can say that innovation is shaped through actions produced by the municipal institution, which, in a non-conflictual way, promotes partial breaks with predefined structures—which belong to the *regime*—by redefining some management schemes, protocols, and so on. This affects some established approaches and practices in exciting forms, igniting a potential change in the governance (and process) to cultural policies that reshape from rigid to fluid, increasing the anti-fragile nature of public policies-practices. The elimination of some steps, the simplification of interaction, etc., appears spontaneously and intuitively in those who govern or manage. Erasing some traits of rigidity of the structure produces a more significant (for people) involvement in the sphere of cultural actions and certain vitalisation of the urban space. This approach allows us to imagine a genuinely profound transformation if pursued structurally. The experimentation could be even more systemic and, in this sense, touch the value dimension, that is to say, the *(land) scape*.

## Conclusions

This article provides an overview of cultural policies and their relation to urban regeneration. Through the lesson learnt from the Issy case, we would try to answer what contributes to define the transitional potential of a cultural policy at an urban scale, having used the empirical perspective of the large panel of cultural initiatives settled in the city. Considering the specificity of the context, we can suggest, to some extent, indications of what further factors would enable the generation of transformative dynamics.

Before, it is necessary to point out again that we are here mostly observing an extraordinary context from a particular point of view. It is a point of view of those who, traditionally, are actors who interact with the *regime* dimension in governance-institutional niches (quoting again Geels). The perspective described here is undoubtedly specific and limited (the research did not independently survey to understand the inhabitants’ response or other forms of independent consultation). Nevertheless, this knowledge from inside the decision-making processes and the governance practices allows a precise reflection on some internal innovation dynamics and relational aspects, eventually tackling these policies’ value dimensions. The specificity of Issy rests on high investments from several perspectives: financial, structural, personnel, the variety of the offer and targets, the scale of the projects, the typologies of network and partnership, etc. Considering the small-to-medium scale of the city/community it represents, it seems possible to observe a series of experimental attitudes and behaviours: the *trust-and-affiliation trait and the loosening-and-flexibility features in the organisation and management of services.* In this sense, Issy-les-Moulineaux represents a veritable laboratory where big and small experiments are conducted in a protected environment (Smith Raven 2012). To be further developed as a veritable experimental laboratory, three hinted directions in work are proposed: one impacts the conduction of the testing process, the second, the content of the experiment, which would be ‘the relation between cultural policies and space’, the third, the role that the Issy environment could play at a broader scale.

The first direction considers the cultural policy environment of Issy as an arena of experimentation. Sustainability transition management (Loorbach et al. 2015) could be a source of inspiration to strengthen integration among citizens and institutions. Following the case study, the inaugurated open spaces/activities by the Clavim association could valorise the self-organisation of groups and activities, conceiving these as part of a more comprehensive transition experiment. Citizens could be integrated with experts from the Municipality in redefining rules together, identifying transition goals within activities, intensifying dialogue and monitoring the process. This is somehow already happening and could benefit from contamination with transition management tools and methodologies.

The second deals with the lines of development for cultural policies in dense urban contexts: a more functional design of “the tailored spatial proximity for a cultural environment”. Issy, among other cities, is a veritable model in terms of proximity of services, and in this respect, we could amplify the role of culture within a discourse about proximity. We could focus on the spatial outcomes of this approach, identifying what kind of spaces we can imagine if we prioritise everyday accessibility to cultural facilities, and asking which effects this produce at the scale of the whole municipality in terms of management and governance of proximity cultural spaces. We are also sure that technology is something prioritised in Issy. It would be interesting to verify to which extent technology supports a shift towards an ordinary/everyday life approach to culture. The results could range from the identification of proper tools, the design of space, the management and the creation of new rules and agreements.

The third direction reflects the replication and networking of the Issy model. This case study is undoubtedly not easily replicable elsewhere. Nevertheless, for this uniqueness, transition dynamics can be much more easily pushed to verify outputs and effectiveness. The complexity of the system is indeed reduced. There is another aspect of interest: so-far-described dynamics could scale up and interact with other situations and environments. From this point of view, the City of Issy could take action with a leading role for a wider territorial sector or network (for example, in the metropolitan area). Other cultural programs and initiatives could increase by taking charge and spreading a culture of inclusiveness, tackling diversity in urban social contexts. This would counterbalance socio-economic and, more relevantly in this dissertation, cultural disparities as they exist in the Paris region. Innovation and technology investors affiliated with the City, could profit from this front role that the Issy-les-Moulineaux could play in a fairer approach to sustainability, that is, the one pointed at through transition studies and transition best practices, balanced by the respect of fragile contexts.

## Data Availability

The datasets generated and analysed during the current study are available in different public repositories, as indicated in the Reference section.

## Footnotes

1 This interaction, built at a distance, is based on sharing a solid background linked to the European research MESOC (Measuring the Social Impact of Culture-Horizon 2020), within which the authors converge.

2 The goals of the WSs were to identify the general aim of the research work, share bibliography and theoretical references (1st WS), build a solid shared database of initiative, policies and data about the case study (2nd WS), identify the primary outcomes and discussion about the core idea of the paper, like the role of cultural policies as a vehicle for socio-spatial transition (3rd WS), carry out further in-depth research on some specific policies developed by the City (4th WS), verify some hypothesis and conclusions with an external expert (5th WS).

3 The authors worked with Esther Destrés, who interacts with the MESOC project for *Relais Culture Europe* and provides links to the data set, websites, articles and documentation about Issy-les-Moulineaux; moreover, she interacts with the authors and facilitates the interview with a local stakeholder. Then Clavim association and in particular Sébastien Masson, in charge of the *Temps des Cerises*, provides information and documents about the association through a dedicated WS-interview and further exchanges. Anna Moro conducts a general interview about the metropolitan strategies and policies from an urban perspective with Matteo Satta, Consultant on EU Affairs and Smart Cities and expert on urban governance and policies in the Parisien Metropolitan area.

4 For example, we consulted various websites of real estate agencies to gather information about costs per sqm in the city. Maps and graphs are the results of our re-elaboration of pre-existing data.

5 Considering data from real estate societies medium prices are around 9500 €/sqm (December 2021- Source: Le Figarò Immobilier https://immobilier.lefigaro.fr/prix-immobilier/issy-les-moulineaux/ville-92040), between 8900 and 9700 (according to Meilleurs Agents: http://www.meilleursagents.com/prix-immobilier/issy-les-moulineaux-92130/).

6 Source: Elaboration by “Linternaute.com” from Observatoire national de la délinquance et des réponses pénales (ONDRP) (https://www.linternaute.com/actualite/delinquance/issy-les-moulineaux/ville-92040).

7 For example, in October 2021, schools profit by an exhibition on models of Leonardo da Vinci (in Temps de Cerises) with dedicated activities, as agreed with the current inspector in charge: the so-called “Educational Paths”, a festival within the framework of Micro-Folies, a history-of-art meeting related to RMN Grand Palais and so on (https://rmngp.fr/linstitution).

8 Considering the subtraction of costs for local police, cameras and surveillance in favour of culture we could reflect on the effect this has on the perception of security, as shown in the result of the “Consultation Citoyenne”, where 10% of the participants are interested in greater control. However, the statistics (as described in paragraph 4.1) inform us of a highly safe context in terms of crime rate.

9 On the 20th of September 2021 the Municipality organized a meeting where Prof. Carlos Moreno explained to the population of Issy his vision.

10 An example is the recent creation of an app, called “CityGem”, a guide about the history of this large park in the heart of the St. Germain Island, with a 2.3 km circuit to discover a flexible and ecological green area.

11 Around the 10% of the population, i.e. 6855 inhabiting or working at Issy-les-Moulineaux answered the survey, i.e. nearly 20% of households of the city.

12 Here is a list of choice of the answers that gets a high appreciation from the citizens (percentage). “Improvements to green spaces 18%. More public transport, extension of line 12: 10%. Develop more cycle paths, bicycle plan/Develop bicycle and two-wheel parking lots: 8%. Improve, make traffic more fluid/Limit traffic cars and utility vehicles crossing the city: 4%. Have more local shops: 10%. Carry out more actions for security, strengthen it: physical surveillance (police, municipal police, police station)/video surveillance: 10%”, Citizen conversations d'Issy-les-Moulineaux December 2020 (https://conversations.issy.com).

13 Thanks to the “Jardiner ma ville” tool, it is possible for residents to garden, maintain and enhance abandoned public spaces, tree bases and planters, but also to propose the establishment of shared vegetable gardens. Five sites exist in Issy-les-Moulineaux: Chemin Bertelotte, a green space cultivated by the association Les Jardins d'Issy; at the corner of rue Gambetta and rue Victor Hugo, an individual maintains a small green space; rue du Viaduc, an individual maintains the base of a tree, in square Blériot, unanchored bins are maintained by the association Issy en Herbe; at Fort d'Issy, a parcel is maintained by the Incroyables Comestibles association.

14 A recent Municipal order to reduce the speed limit to 30 km/h on May 14, 2020, in the majority of the urban local streets, source: https://www.issy.com/actualites/mesures-circulation.

15 At 700 m from the *Ecoquartier*, the new factory automatically collects the waste produced by the new district thanks to the pneumatic collection.

16 The entire description of the project can be read at this address, a webpage managed by the Minister of Ecological Transition: http://www.ecoquartiers.logement.gouv.fr/operation/1027/.

17 The interview with the responsible for *Borde de Seine Ecoquartier* can be found at www.youtube.com/watch?v=UWhehrXkOVw.

18 An example is the Intelligent Cities Challenge (ICC), an European Commission initiative bringing together 136 cities to achieve intelligent, socially responsible and sustainable growth through advanced technologies. For further information see: https://www.intelligentcitieschallenge.eu/about-intelligent-cities-challenge.
